# Research on Data Poisoning Attack against Smart Grid Cyber–Physical System Based on Edge Computing

**DOI:** 10.3390/s23094509

**Published:** 2023-05-05

**Authors:** Yanxu Zhu, Hong Wen, Runhui Zhao, Yixin Jiang, Qiang Liu, Peng Zhang

**Affiliations:** 1 School of Aeronautics and Astronautics, University of Electronic Science and Technology of China, Chengdu 611731, China; 2 Aircraft Swarm Intelligent Sensing and Cooperative Control Key Laboratory of Sichuan Province, Chengdu 611731, China; 3 Electric Power Research Institute, China Southern Power Grid Co., Ltd., Guangzhou 510663, China

**Keywords:** smart grid, edge computing, poisoning attack, regression task, online learning

## Abstract

Data poisoning attack is a well-known attack against machine learning models, where malicious attackers contaminate the training data to manipulate critical models and predictive outcomes by masquerading as terminal devices. As this type of attack can be fatal to the operation of a smart grid, addressing data poisoning is of utmost importance. However, this attack requires solving an expensive two-level optimization problem, which can be challenging to implement in resource-constrained edge environments of the smart grid. To mitigate this issue, it is crucial to enhance efficiency and reduce the costs of the attack. This paper proposes an online data poisoning attack framework based on the online regression task model. The framework achieves the goal of manipulating the model by polluting the sample data stream that arrives at the cache incrementally. Furthermore, a point selection strategy based on sample loss is proposed in this framework. Compared to the traditional random point selection strategy, this strategy makes the attack more targeted, thereby enhancing the attack’s efficiency. Additionally, a batch-polluting strategy is proposed in this paper, which synchronously updates the poisoning points based on the direction of gradient ascent. This strategy reduces the number of iterations required for inner optimization and thus reduces the time overhead. Finally, multiple experiments are conducted to compare the proposed method with the baseline method, and the evaluation index of loss over time is proposed to demonstrate the effectiveness of the method. The results show that the proposed method outperforms the existing baseline method in both attack effectiveness and overhead.

## 1. Introduction

The construction of a power grid requires a large number of terminal devices, and the popularity of these devices has made the amount of data on the grid exponentially increase. However, with the increasing number of network structures and the growing number of nodes, the security and real-time performance of the power grid have been greatly challenged while promoting the smart grid [1]. When the network architecture based on the cloud center is faced with a large number of node devices and continuous data, the way of remote communication cannot meet the requirements of the speed bandwidth of the grid. For some time-sensitive tasks of the smart grid, the cloud center can easily reach the bottleneck of the processing capacity. To solve this problem, it is very effective to introduce edge computing in the smart grid [2,3]. Edge computing has unique advantages such as data localization and edge intelligence [4], which not only reduces the time cost for data to reach the cloud, but also enables data processing directly through the edge, thereby reducing communication and computing pressure in the cloud center [5]. However, the advent of edge computing also presents some security challenges. Because of thew less potent security protocols in the resource-constrained edge hardware [6], malicious attackers can invade edge nodes through these terminal devices. At the same time, due to the recent trend of edge intelligence [7,8,9], more and more computing and storage tasks are performed on edge nodes [10,11], so they are vulnerable to malicious network attacks, such as masquerade and spoofing attacks [12,13], data and model poisoning, and evasion attacks [6]. Data poisoning [14] is a typical attack against machine learning models. Malicious attackers pollute training data to manipulate important models and predictive results by disguising as terminal devices, which could be fatal to the operation of a smart grid, causing issues such as large-scale power outages, power market price disruption, and so on. Unfortunately, current research on the data security of the smart grid mainly focuses on false data injection attacks (FDIA) [15,16,17,18] rather than poisoning attacks on machine learning models in edge computing environments. However, compared to traditional FDIA attacks, poisoning attacks are more covert and harmful to the smart grid.

Currently, data poisoning attack research primarily focuses on image processing or classification scenarios, with limited attention being given to regression tasks. Furthermore, the target model is typically trained in an offline environment instead of undergoing online training. In the context of the smart grid, most machine learning tasks involve the prediction of continuous power data through regression instead of classification, and many machine learning algorithms are trained using an online learning process. As such, there is a need for online training, where machine learning models are updated based on new incoming data. However, the existing research on poisoning attacks is not suitable for the edge computing environment of the smart grid. In addition, the computationally expensive nature of poisoning attacks hinders their direct application in resource-constrained edge computing environments in the smart grid. It is necessary to optimize the attack framework and strategies to enhance efficiency and reduce computational overhead. Therefore, the main contributions of this work are as follows:

- It proposes an online poisoning attack framework based on the online regression task model and applies it to the edge computing environment of the smart grid for the first time. In contrast to traditional offline attacks, the framework achieves the goal of manipulating the model by incrementally polluting the sample data stream that arrives at the appropriately sized cache to optimize the efficiency of the attack.
- It proposes a point selection strategy based on sample loss. Compared to the traditional random point selection strategy, this strategy makes the attack more targeted, thereby enhancing the attack’s efficiency.
- It proposes a batch-polluting strategy to update batch poisoning points based on the direction of gradient ascent synchronously. This strategy reduces the number of iterations required for inner optimization and thus reduces the time overhead.
- It implements online gray-box poisoning attack algorithms with the framework and strategies mentioned above. It evaluates the effectiveness and overhead of the proposed attack on edge devices of the smart grid using an online data stream simulated with offline open-grid datasets.

The rest of this paper is organized as follows. Section 2 presents the related work on data poisoning attack. Section 3 proposes an online incremental poisoning attack framework for the online regression learning task in the edge computing environment of the smart grid. Section 4 introduces online algorithms for gray-box poisoning attack. Section 5 presents the experiment and result analysis. Finally, Section 6 concludes this paper and provides the future research directions.

## 2. Related Works

The recent literature has demonstrated a significant decrease in the performance of learning models when training data is poisoned [19,20,21,22]. Data poisoning enables attackers to manipulate prediction models, thereby disrupting the automated decision-making process. For instance, Vrablecova et al. propose a load forecasting model for the smart grid [23]. If attackers manipulate the forecasting model to lower the forecasted demand for electricity in a specific period, this can result in power outages due to a shortage of the electricity supply. Conversely, if the forecasted demand is higher than the actual demand, this can lead to excess power, overloading the power distribution system. Therefore, compared to traditional FDIA attacks, data poisoning attacks are more insidious and pose a greater threat to smart grid.

Data poisoning attacks can be classified into three categories based on the adversary’s knowledge of the target prediction model: white-box, black-box, and gray-box attacks. In a white-box attack scenario, attackers have complete knowledge of five factors: feature space, learning type (e.g., DNN or SVM), learning algorithm, learning hyperparameters, and training datasets [24]. A black-box attack scenario is opposite to a white-box one, assuming that attackers have no knowledge of all of the five factors already described previously. In the real world, absolute black-box and white-box attacks usually do not exist. The reasonable assumption is that attackers can know at least part of the training data or surrogate model [25] (also called the substitute model [26]) by model stealing, which is a kind of gray-box attack [24,25,27]. In addition, attackers can only gain partial access to the segments of the sample stream in the scenario of online attacks, which falls within the realm of gray-box attacks.

Based on the poisoning area of training samples, existing data poisoning attacks can be categorized into three types: label poisoning, feature poisoning, and sample poisoning. In label poisoning, the label vector ***y*** of a training sample is corrupted. In feature poisoning, the feature matrix ***X*** of a training sample is corrupted. In sample poisoning, both ***X*** and ***y*** of a training sample are corrupted. The implementation of label poisoning is simpler than that of feature poisoning. Biggio et al. [28] assume that attackers can control some training data, and aim to disturb the support vector machines (SVMs)’ learning process. It introduces label noise to training points for impacting the discriminative model learned from the training data deleteriously, which indicates that this attack does have an attack effect on the machine learning model. Xiao et al. [29] evaluate the security of SVMs against well-crafted adversarial label noise attacks, which aim to maximize the classification error of a SVM by flipping multiple labels in the training data. In order to analyze the effectiveness of the considered attacks, they carry out a large number of experiments on both linear and nonlinear SVM models. Paudice et al. [30] develop the heuristic to craft efficient label flipping attacks, of which experiments show the effectiveness at mitigating the effect of label flipping attacks on a linear classifier. Ample evidence suggests that poisoning the feature matrix ***X*** is still the most effective approach, compared to modifying the label ***y*** alone in label flip attacks. Therefore, we are more concerned with contamination of the feature matrix ***X***.

In terms of feature poisoning, Biggio et al. [31] propose a poisoning attack to tamper with the features of some samples using a gradient ascent strategy in which the gradient is computed based on the model parameters of the support vector machine, the selected loss function and the characteristics of input samples. Experiments show that the gradient ascent procedure has a very significant impact on the classification accuracy of a SVM. Burkard and Lagesse [32] propose poisoning attacks on a SVM that is learning from data stream. In addition to the SVM model, researchers have proposed many attack methods against other machine learning models. Mei et al. [33] present that optimal training set attack can be formulated as a bi-level optimization problem, which can be solved using gradient methods with certain Karush–Kuhn–Tucker conditions, and demonstrate the effectiveness of the method in support vector machines with extensive experiments. In 2016, Zhu et al. [34] constructed poisoning attack methods for the class of linear autoregressive models. In 2017, KOH and Liang [35] developed a form of efficient attack that only requires oracle access to gradients and Hessian-vector products, which is useful for multiple purposes in linear models and convolutional neural networks. In the same year, Battista Biggio et al. [36] proposed a novel poisoning attack based on the idea of back-gradient optimization, which constructs poisoning samples against the regression model. Cisse et al. [37] proposed a poisoning attack named Houdini on the image classification network, which was proven to be effective for cheating the speech recognition model. In 2019, Chen and Zhu [38] presented the optimal attack using the linear quadratic regulator (LQR) for linear models, and model predictive control (MPC) for nonlinear models, which are effective in the black-box setting. Li et al. [39] introduced a data poisoning attack on collaborative filtering systems, showing that attackers with full knowledge of the target model can build malicious data to maximize the attack target, and imitate normal user behavior to avoid being detected.

According to the different poisoning strategies, there are three categories: label flipping poisoning attack, gradient-ascent poisoning attack and statistically based poisoning attack. The first method constructs a poisoned sample by flipping the target value of ***X*** or ***y*** to the other side of the feasibility domain [40]. The second method uses gradient ascent to iteratively update training points and stop at the convergence, where they obtain a poisoned sample [26]. The third method generates a poisoned sample from a multivariate normal distribution with the mean and covariance estimated from the training data [19]. In general, although label flipping and statistical-based methods are relatively low-cost, these two methods create poisoning samples that are easily detected and discarded by human examiners or automated detectors. The gradient ascent method is the most computationally expensive method, but it is the most effective and confidential.

Table 1 summarizes the various poisoning attack methodologies outlined above. Most of the research mentioned above is focused on image processing or classification scenarios, but little of it is focused on regression tasks. Although Jagielski and Biggio et al. [19,33,36] have proposed possible attack methods against the regression model, the training and poisoning of the methods occur in an offline environment instead of online. Zhang et al. [41] provided the latest research on online learning attacks. Their study provides an optimization control method for poisoning attacks on nonlinear classification models in the black-box mode. Wang et al. [42] presented heuristic attacks against the binary classification with an online gradient descent learner, which is more like the clairvoyant online attacks (with full knowledge of future samples) mentioned in that study. Inspired by these two papers, we apply online poisoning to optimize attacks on regression models.

The core issue of data poisoning attack is the expensive bi-level optimization problem, which has a time complexity of $O(iter\_num*n*k^{3})$ in the offline mode. Here, n is the total number of samples, k is the dimension of features, and iter_num refers to the rounds of updating poisoned sample features according to the gradient ascent’s direction. Therefore, existing research mainly focuses on exploration based on the above factors. Koh et al. [43,44,45] attempted to find the sample set with the largest impact on the model and applied it to attacks. Their method is based on a binary classification model, and their conclusion is that the sample points with the greatest impact on binary classification models can be reduced to two non-repetitive samples. However, these two sample points are not unique, so the sample size is not essentially reduced. Inspired by this study, this paper focuses on the influence of sample selection on attack efficiency and applies it to regression models. Memon et al. [46] studied the impact of the minimum number of samples on model training, and affirmed Green’s sample size principle (Green (1991) [47] recommends N≥50+8 m, where N and m are the minimum number of samples and the number of parameters in the model, respectively), but did not provide a definitive conclusion. There are also other heuristic methods that bypass the bi-level optimization problem, such as the statistically based methods and label flipping-based methods mentioned above. While more straightforward heuristic attacks [48,49,50] have shown promising results in terms of attack effectiveness and cost optimization, their applicability and robustness are still too limited in the presence of suitable defense mechanisms. Other promising research approaches include building upon the foundations of other research areas, such as meta-learning and hyperparameter optimization, which continuously develop more effective techniques to solve the double-layered problem involving learning algorithms. However, there has been little substantive progress in this area so far.

In summary, this paper focuses on the gray-box attack model against the online regression model using a gradient ascent-based poisoning strategy in the edge environment. We have re-examined the traditional attack model and proposed a novel framework for online attacks that is designed to better suit the resource-constrained environment of the smart grid. Our approach focuses on optimizing selection points and polluting strategies to achieve a more efficient and cost-effective poisoning of sample features.

## 3. Attack Model

The objective function of a machine learning model is a necessary condition for data poisoning attacks. We design the poisoning attack algorithm based on the objective function to construct poisoned samples that can have the greatest impact on the model’s performance. The definitions of the symbols used in this section are shown in Table 2. We assume that the objective function being attacked of the linear regression model is (1)

(1) $$y=h(X)=\theta_{1}x_{1}+\theta_{2}x_{2}+\cdots +\theta_{k}x_{k}=\theta^{T}X,\;\theta ={\left[\theta_{1},\theta_{2},\cdots ,\theta_{k}\right]}^{T},X={\left[\begin{array}{ccc} x_{11} & \cdots & x_{n1}\\ \vdots & \ddots & \vdots \\ x_{1k} & \cdots & x_{nk} \end{array}\right]}_{k\times n},y=[y_{1},y_{2},\cdots ,y_{n}]$$

Let θ denote parameter vector, ***y*** is label vector. X denotes feature matrix of the model, of which subscript k and n represent the feature dimension and number of samples, respectively. Loss function is denoted by Mean Square Error (MSE):

(2) $$J(D_{s},\theta )=\frac{1}{n}\sum_{i=1}^{n}{\left(h(x_{i})-y_{i}\right)}^{2}$$

As in (2), assume the original input samples is $D_{s}={\left\{(x_{i},y_{i})\right\}}_{i=1}^{n}$, in which $x_{i}$ denotes the feature vector and $y_{i}$ denotes the response label variable of $x_{i}$. The normal training goal is to calculate the optimal parameter, $\theta^{*}$, for the minimum loss function shown in (3). In the edge computing environment, $D_{s}$ represents samples cached in smart-grid edge devices, and n represents the capacity of the cache to store samples. The process of calculating parameter $\theta^{*}$ is usually iterative, such as the gradient descent method given in is (4). According to the iteration frequency, it can be divided into three methods: SGD (stochastic gradient descent), MBGD (mini-batch gradient descent) and BGD (batch gradient descent). For the BGD method, the parameters of $\theta_{i}$ are iterated only once through all samples. For the SGD method, parameters are iterated once for each sample, while for the MBGD method, parameters are iterated once for each batch size sample. SGD can quickly converge under the condition of less computing resources, which is more suitable for a resource-constrained environment in edge computing.

(3) $$\theta^{*}=argminJ(D_{s},\theta )$$

(4) $$\theta_{i+1}=\theta_{i}-\alpha \nabla_{\theta_{i}}J(D_{s},\theta_{i})$$

**Figure 1:**
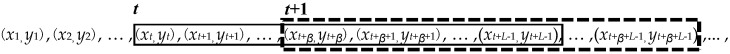
Cache strategy of samples.

In order to alleviate the catastrophic forgetting problem [51] in incremental learning, we define a cache strategy similar to that of the sliding window. Figure 1 illustrates the strategy of storing the online training sample stream arriving at the edge node at time t and time t+1. In Figure 1, the solid and dashed boxes represent the samples cached at time t and time t+1, respectively. L denotes the capacity of the cache. β denotes the number of samples being covered (forgotten). According to this strategy, at time t, the sample $D_{s}^{\left(t\right)}={\left\{(x_{i},y_{i})\right\}}_{i=t}^{t+L}$. The parameter β is used to control the degree of forgetting for the training data on the edge node. The new samples completely overwrite the historical samples from the previous time step when β=L, while β=0 means that the new samples do not overwrite any historical samples from the previous time step.

Building upon the above strategy, this paper assumes that attackers can manipulate the $D_{s}$ and inject malicious data to poison the learning process, or use attack points to subvert the online learning process, when the training data is received sequentially. This section will propose a gray poisoning attack model for the regression task of online learning.

We define our adversary model following the framework proposed in [29], which involves identifying the adversary’s goals and describing their knowledge and capabilities. This information is then utilized to define an optimal attack strategy.

### 3.1. Adversary’s Goal

The objective of attacks can be defined as three types of integrity, availability, or privacy violation [21], of which specificity can be targeted or indiscriminate [52,53]. The integrity attack aims to selectively poison specific samples to cause particular mis-predictions, while the availability attack aims to indiscriminately corrupt learning models by poisoning training samples. The goal of our paper falls into the first type, which is to compromise the integrity of the model by changing its parameters to the greatest extent possible, without being detected by the target model. In the case of online learning, the attack objective is to inject malicious data into the training stream to maximize the loss function value of the model at the end of training.

### 3.2. Adversary’s Knowledge

In this paper, we consider a gray-box attack method for poisoning, where the attacker is assumed to have knowledge of the learning-type (regression) learning algorithm, L, and partial training samples, $D_{s}^{\left(t\right)}$, but does not know the trained parameters. In an online learning environment at the edge, training samples arrive at the edge’s cache one after another, making it impossible for the attacker to know the entire training sample set. The attacker can only access the samples, $D_{s}^{\left(t\right)}$, in the cache. In the extreme case, the attacker does not even know anything about the training samples, but fortunately can construct substitute datasets [21,36], from which trained parameters can be estimated by optimizing the learning algorithm.

### 3.3. Adversary’s Capability

The attacker’s capability is limited to crafting the training sample data; i.e., altering the training process is not allowed. We also assume that the attacker has full control of the training data steam including feature or label values at a certain time point. However, there is a maximum limit to the number of $n_{p}$ samples in the data stream that can only be changed, under which condition the target model incrementally trains the modified data stream of polluted samples not caught by the anomaly defense mechanism. We define the poisoning rate, γ, as the actual fraction of the training stream controlled by the attacker. Let us assume that $n_{p}^{\left(i\right)}$ samples are poisoned at time i, and that the total number of poisoning samples $n_{p}=\sum_{i=1}^{t}n_{p}^{\left(i\right)}$ at the end of time t. The poisoning rate $\gamma =(\sum_{i=1}^{t}n_{p}^{\left(i\right)})$*/tL* at time t. This paper assumes that the same number of sample points are poisoned at each time, so it is easy to prove that $\gamma =n_{p}^{\left(i\right)}$*/L*.

### 3.4. Adversary’s Strategy

In the case of online learning, the strategy of attack is to maximize the prediction error by the training sample stream at the time t instead of offline datasets. Therefore, the bi-level optimization problem [19,33,36,40] for the offline condition is no longer applicable online. The attacker’s strategy is formulated as an online incremental bi-level optimization strategy, which can be written as (5) and (6):

(5) $${argmax}_{D_{s}^{t}}J(D_{s}^{\left(t\right)}(or{D\prime }_{s}^{\left(t\right)}),\theta_{p}^{*}),$$

(6) $$s.t.\theta_{p}^{*}\in {argmin}_{\theta_{t-1}}L({D_{s}^{\left(t-1\right)}(or{D\prime }_{s}^{\left(t-1\right)})\cup D_{p}^{\left(t-1\right)},\theta }_{t-1})$$

Equation (6) is called inner optimization, corresponding to retraining the regression algorithm, L**,** on both the clean training samples, $D_{s}^{\left(t-1\right)}$, and the poisoned samples, $D_{p}^{\left(t-1\right)}$, at time t−1, which generates the optimal parameter $\theta_{p}^{*}$. Equation (5) is called outer optimization, which amounts to selecting the poisoned points, $D_{p}^{\left(t-1\right)}$, to maximize the loss function, J, on the untainted data set, $D_{s}^{\left(t\right)}$ (which does not contain any poisoning points), at time t. $D_{s}^{\left(t-1\right)}$ and $D_{s}^{\left(t\right)}$ denote training samples stored in the cache at the adjacent time. $\theta_{p}^{*}$ can also be estimated using the substitute training data stream’s $D_{s}^{\prime }$ instead of $D_{s}$. The above online attack strategy is not one of one-off poisoning, but continues to poison samples reaching the cache as time progresses. It is also possible to set the frequency of poisoning, such as to poisoning at random or in regular intervals.

**Figure 2:**
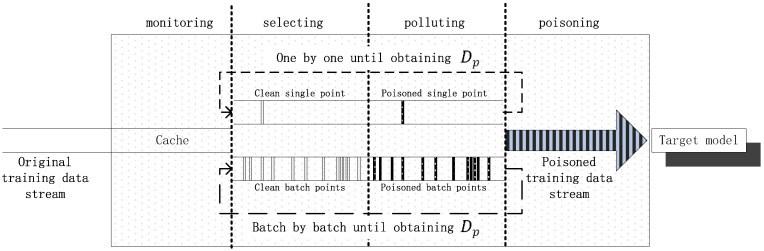
Schematic diagram of attack.

**Figure 3:**
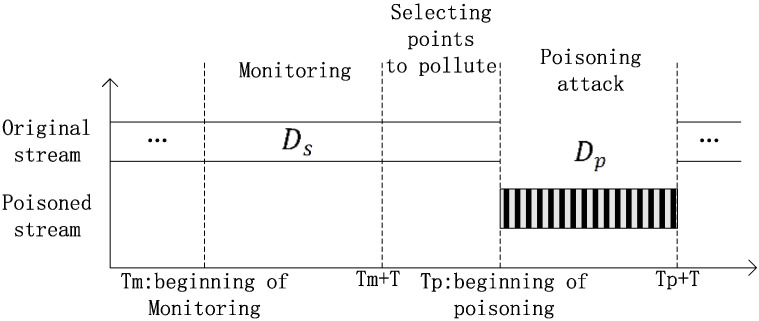
Schematic diagram of poisoning process during online monitoring, selection, pollution and poisoning of sample points.

Figure 2 illustrates the principle of online poisoning attacks, which consists of four stages: samples monitoring, attack point selection, data polluting, and steam poisoning. Each stage is separated by a dashed line, as shown in the rectangular box. During the monitoring stage, a segment of the length, *L*, is read from the original sample data stream and stored in the cache. Two strategies are employed in the selection and polluting stages: one is to select and pollute one point at a time until obtaining a poison sample, $D_{p}$, of size $n_{p}$, and the other is to select and pollute points in batches until obtaining a poison sample, $D_{p}$. The light-colored vertical bars in the selecting stage represent the selected clean sample points, and the dark-colored vertical bars in the polluting stage represent the contaminated sample points. The selection and polluting stages are iteratively executed, as indicated by the long dashed line with arrows. The poisoned stream with dark striped arrows in the poisoning stage represents the $D_{p}$ inserted into the original data stream, which is then sent to the target model, indicating the completion of the entire poisoning attack. Figure 3 provides an overall view of the attack process, where the dark striped rectangular bar represents the poisoned stream mixed into the normal data stream and the horizontal axis represents the temporal relationship of the stream. The poisoning attack can be executed repeatedly or separately in intervals.

## 4. Attack Algorithm

**Figure 4:**
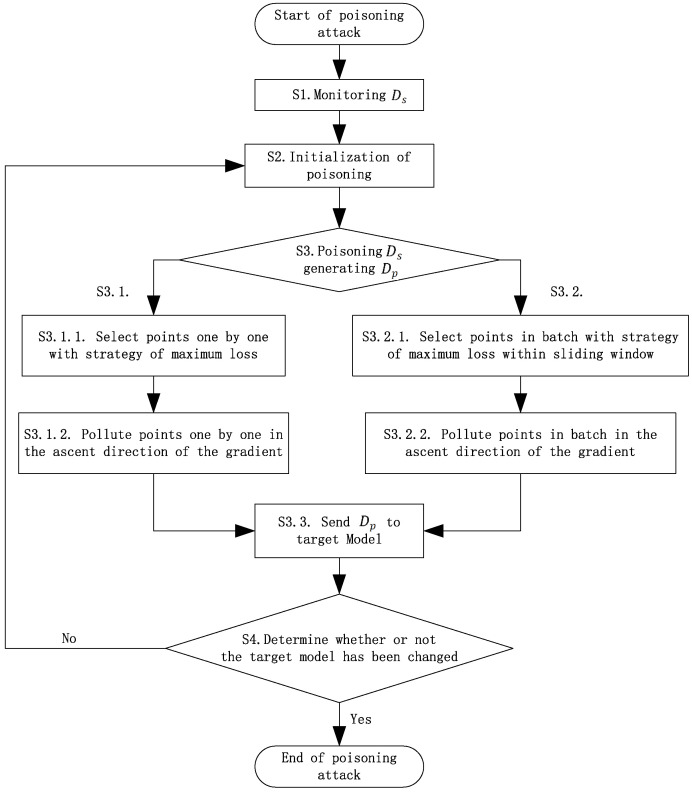
Flowchart of attack.

Figure 4 presents the flowchart of the online poisoning attack. In step S1, a certain number of online training samples are obtained during the monitoring of the original stream. After the initialization of step S2, step S3 selects sample data points with a certain poisoning rate from the stream based on the strategy of maximum loss and pollutes these points using the gradient ascent strategy. There are two strategies to select data points from to pollute: single-point selection and batch-point selection from a sliding window, which determine whether or not to pollute a sample point or a batch of sample points, respectively. The polluting operation will update the selected sample points to new values according to the gradient ascent strategy and learning step size. Based on the arithmetic used in the poisoning attack, the selecting and polluting operations will take a certain amount of time, during which the original sample data stream is continuously input into the target model. Once the selecting and poisoning operations are completed, the poisoned data stream is injected into the original training stream being sent to the target model. This section describes the online poisoning attack algorithm step by step. The definitions of the symbols used in this section are shown in Table 2.

Step S1 obtains a certain number of online training samples, $D_{s}$, during the monitoring of the original training data stream. Samples $D_{s}^{t}$ and $D_{s}^{t+1}$ from the adjacent time are saved in the cache as inputs in the attack algorithm. From time zero, training samples arrive one after another, and the model is trained iteratively. When the model reaches a convergence state at time t, the trained parameter $\theta^{\left(t\right)}$ is obtained as the initial parameter of the attack method (cf. line 1 to 4 in Algorithms 1 and 2).

Step S2. Initialize the maximum poisoning sample number, q=γ∗L, the cache size, L, of the data stream, $D_{s}$ (L is greater than q), the width of the slide window, m (according to the cache size, *L*, in the $D_{s}$ and the number of poisoned samples, q, m<q) (cf. line 5 in Algorithms 1 and 2).

Step S3. Select and pollute points from the $D_{s}$ to generate poisoned samples, $D_{p}$, with two strategies: single-point (S3.1) and batch-point (S3.2).

Step S3.1. Pollute points based on single-point selection, and the specific implementation process is shown in Algorithm 1:
**Algorithm 1** Online poisoning attack based on single-point strategy (abbreviated as ODPA-SP)**Input:** training data stream, $D_{s}$, L,J, positive constant,ε (or ${D^{\prime }}_{s}$ for the black-box mode), poisoning rate, γ, and cache size, *L*
1: t←0(Initialization of time t)
2: **repeat**
3:   t←t+1
4: $until\;{(\theta }^{\left(t\right)}\leftarrow {argmin}_{\theta }L(D_{s}^{\left(t\right)},\theta ))$ (initialization of model)
5: q←γ∗L (initialization of poisoned point number)
6: $indices\leftarrow sort({\left\{J(D_{s}^{\left(i\right)},\theta^{\left(t\right)})\right\}}_{i=t}^{t+L-1})[:q]$
7:$D_{p}^{\left(t\right)}\leftarrow (x[indices],y[indices])$
8: repeat
9:  $J^{\left(t\right)}\leftarrow J(D_{s}^{\left(t+1\right)},\theta^{\left(t\right)})$
10:  for c=1,⋯,q **do**
11:   $x_{c}^{\left(t+1\right)}\leftarrow \Pi (x_{c}^{\left(t\right)}+\alpha \nabla_{x_{c}^{\left(t\right)}}J(D_{s}^{\left(t\right)},\theta^{\left(t\right)}))$
12:   $\theta^{\left(t+1\right)}\leftarrow {argmin}_{\theta^{\left(t\right)}}L(D_{s}^{\left(t\right)}\cup \{(x_{c}^{\left(t+1\right)},y_{c})\},\theta^{\left(t\right)})$
13:   $J^{\left(t+1\right)}\leftarrow J(D_{s}^{\left(t+1\right)},\theta^{\left(t+1\right)})$
14:  **end for**
15:  send the poisoned samples, $D_{p}^{\left(t\right)}$, to the target model
16:  $indices\leftarrow sort({\left\{J(D_{s}^{\left(i\right)},\theta^{\left(t+1\right)})\right\}}_{i=t+1}^{t+L})[:q]$
17:  $D_{p}^{\left(t+1\right)}\leftarrow (x[indices],y[indices])$
18:  t←t+1
19:  **until** $\left|J^{\left(t\right)}-J^{\left(t-1\right)}\right|<\varepsilon$

Step S3.1.1. Traverse samples of $D_{s}^{\left(t\right)}$, calculate the loss function value according to Formula (7) and sort the loss from large to small, selecting the first q sample points as the initial poisoning sample points (Algorithm 1, line 6 to 7). This selection strategy is established based on the following observation: sample points with a larger loss in the current model have a greater influence on the model, which is grounded on the fact that points with higher loss are typically situated on the decision boundary. Formula (7) provides the method for computing the loss for each point, where $\theta^{\left(t\right)}$ represents the initial model parameters trained using the fragments of the sample stream stored in the cache.

(7) $$J(D_{s}^{\left(i\right)},\theta^{\left(t\right)})=\frac{1}{2}{\left(h(x_{i})-y_{i}\right)}^{2}$$

Step S3.1.2. For each point in $D_{p}^{\left(t\right)}$, $x_{c}$ is updated (according to Formula (8)) through the ascent direction of the gradient $\nabla_{x_{c}}J(D_{s}^{\left(t+1\right)},\theta^{\left(t+1\right)})$ to the outer optimization (evaluated by Formula (5)). Note that $x_{c}$ should be enforced to lie within the feasible domain (e.g., $x_{c}\in {\left[0,1\right]}^{d}$), which can be typically achieved through simple projection operator Π [21,31,36] (Algorithm 1, line 11). Then, add the poisoned samples $\left\{(x_{c}^{\left(t+1\right)},y_{c})\right\}$ to $D_{s}^{\left(t\right)}$, retraining the model to update the parameter of the inner optimization (evaluated by Formula (6)) (Algorithm 1, line 12).

(8) $$x_{c}=x_{c}+\alpha \nabla_{x_{c}}J(D_{s}^{\left(t+1\right)},\theta^{\left(t+1\right)})$$

The algorithm ODPA-SP in this section does not change the complexity of the traditional offline bi-level optimization algorithm but rather converts it into an online version, while also optimizing the selection strategy and poisoning strategy of the adversarial sample points. The operations used to train the model with the samples in lines 4 to 12 of the algorithm have a computational complexity of $O(k^{3})$, where k is the feature dimension. The loops in lines 8 to 19 implement the iterative steps for updating the poison samples using the gradient ascent method. The loops in lines 10 to 14 update each poison sample point iteratively. Therefore, the computational complexity of the ODPA-SP algorithm remains as $O(iter\_num*n*k^{3})$.

Step S3.2. is to pollute points based on the batch-point selection from the slide window m, and the specific implementation process is shown in Algorithm 2.
**Algorithm 2** Online poisoning attack based on batch-points (abbreviated as ODPA-BP)**Input:** training data stream, $D_{s}$, L,J, positive constant ε (or ${D^{\prime }}_{s}$ for the black-box mode), poisoning rate, γ, cache size, *L*, and size of the sliding window, m
1:t←0 (initialization of time t).
2: **repeat**
3:   t←t+1
4: $until\;{(\theta }^{\left(t\right)}\leftarrow {argmin}_{\theta }L(D_{s}^{\left(t\right)},\theta ))$ (Initialization of model)
5:q←γ∗L (initialization of poison point number)
6: **for** i∈range(t,t+L−1−q,m)  **do**
7:  $indices\leftarrow getmax(J(D_{s}^{\left(i:i+q-1\right)},\theta^{\left(t\right)}))$
8: **end for**
9: $D_{p}^{\left(t\right)}\leftarrow {\left\{x_{i},y_{i}\right\}}_{i=indices}^{indices+q-1}$
10: repeat
11:  $J^{\left(t\right)}\leftarrow J(D_{s}^{\left(t+1\right)},\theta^{\left(t\right)})$
12:  $x_{1:q}^{\left(t+1\right)}\leftarrow \Pi (x_{1:q}^{\left(t\right)}+\alpha \nabla_{x_{1:q}^{\left(t\right)}}J(D_{s}^{\left(t\right)},\theta^{\left(t\right)}))$
13:  $\theta^{\left(t+1\right)}\leftarrow {argmin}_{\theta^{\left(t\right)}}L(D_{s}^{\left(t\right)}\cup {\left\{(x_{i}^{\left(t+1\right)},y_{i})\right\}}_{i=1}^{q},\theta^{\left(t\right)})$
14:  $J^{\left(t+1\right)}\leftarrow J(D_{s}^{\left(t+1\right)},\theta^{\left(t+1\right)})$
15:  send the poisoned samples $D_{p}^{\left(t\right)}$ to target model
16:  **for** i∈range(t+1,t+L−q,m)do
17:   $indices\leftarrow getmax(J(D_{s}^{\left(i:i+q-1\right)},\theta^{\left(t+1\right)}))$
18:  **end for**
19:  $D_{p}^{\left(t\right)}\leftarrow {\left\{x_{i},y_{i}\right\}}_{i=indices}^{indices+q-1}$
20:  t←t+1
21: **until** $\left|J^{\left(t\right)}-J^{\left(t-1\right)}\right|<\varepsilon$


Step S3.2.1. After model initialization, traverse samples of $D_{s}^{\left(t\right)}$ and calculate the loss function value in the sliding window in accordance with Formula (9). Select batch points with the largest loss as the initial poisoning sample points (Algorithm 2, line 6 to 9). This step, instead of computing the loss for each sample point with respect to the initial model, it computes the loss value for a batch of m sample points in the cache according to Formula (8). It traverses the subsets of size m in the cache samples using a sliding window and identify the subset with the maximum loss as the poison sample points.

(9) $$J(D_{s}^{\left(i:i+q-1\right)},\theta^{\left(t\right)})=\frac{1}{q}\sum_{i=t}^{t+q-1}{\left(h(x_{i})-y_{i}\right)}^{2}$$

Step S3.2.2. Update batch points $\left[x_{t},x_{t+1},\cdots ,x_{t+q-1}\right]$ in the ascent direction of the gradient at once in accordance with Formula (9); meanwhile, map these points to lie within the feasible domain through projection operator Π (Algorithm 2, line 12). Then, add these samples ${\left\{(x_{i}^{\left(t+1\right)},y_{i})\right\}}_{i=1}^{q}$ to the $D_{s}^{\left(t\right)}$, retraining model to update the parameter of the inner optimization (evaluated by Formula (6)) (Algorithm 2, line 13). In this step, the algorithm synchronously computes the gradient of the loss function with respect to the q poison sample points according to Formula (10).

(10) $$\left[x_{t},x_{t+1},\cdots ,x_{t+q-1}]=[x_{t},x_{t+1},\cdots ,x_{t+q-1}]+\alpha \nabla_{x_{t:t+q-1}}J(D_{s}^{\left(t+1\right)},\theta^{\left(t+1\right)}\right)$$

Step S3.3. Send the poisoned samples, $D_{p}^{\left(t\right)}$, to the target model when the q sample points have been poisoned, after which new q points are re-selected for the newly arrived samples $D_{p}^{\left(t+1\right)}$ (Algorithm 1, line 15 to 18 and Algorithm 2, line 16 to 20).

Step S4. Repeat or intermittently implement S2 in S4. At the same time, the predicted results of the model are validated, and when the results are biased, this demonstrates the poisoning attack was successful. Repeat the steps above until there is no significant change in the loss function value (Algorithm 1, line 19 and Algorithm 2, line 21).

From the description of algorithms above, it can be seen that compared to offline methods, online methods of poisoning continuously train with new samples incrementally instead of traversing the same offline samples iteratively. Compared to the offline algorithms, the computational complexity of the online algorithm remains unchanged, but the size of the sample for each calculation is reduced. The difference between the ODPA-BP and ODPA-SP algorithms lies in lines 12–13, which reduce the q computations in ODPA-SP to q/m computations, thereby reducing the overall algorithmic overhead.

## 5. Experiments and Analysis

This section evaluates the effectiveness of the proposed online poisoning attack framework, loss-based point selection strategy, and batch-point pollution strategy when applied to edge devices. The specific evaluations for the following questions are conducted:

Question 1: Does the online poisoning attack method have less time overhead compared to the offline method?

Question 2: Can the loss-based point selection strategy improve the effectiveness of the poisoning attack effectively?

Question 3: Can the batch-point-selection poisoning strategy reduce the time overhead effectively?

Question 4: What are the optimal strategies for online single-point poisoning and online batch poisoning attacks under different conditions?

Question 5: What is the actual impact of poisoning attacks on power prediction?

**Experimental setup.** In order to simulate the edge computing environment of the smart grid, the prediction algorithm was run in Linux OS in edge-embedded boards, which were mainly configured with a main chip with a cortex-A7 core, 1.2 GHz, 256 MB RAM, and 512 MB ROM. We developed experiments with python, and processed data with numpy, sklearn, math and pandas libraries. Our code is available at https://github.com/yannickzhu/ODPA.git (accessed on 2 May 2023). The target model used in this experiment is the stochastic gradient descent (SGD) linear regression model, which simulates the process of an online updating model in an edge intelligence environment. The evaluation metrics mainly include MSE loss, the running time of attack, and the loss over time (LOT). The calculation method of MSE loss is to use the poisoned model to predict the test set samples and calculate the MSE loss between the predicted values and the ground truth. The running time of the attack records the time interval from the start of the attack to the end of the attack. The calculation method of LOT is to determine the ratio of the MSE loss to the running time of attack. Compared to the MSE metric, LOT takes into account the factor of time overhead and can provide a more comprehensive evaluation of the effectiveness of the attack. This article uses the OptP method proposed in [19] as the baseline algorithm. The OptP algorithm is a classic offline poisoning attack algorithm that has been widely used as a foundation for many research studies, making it highly representative.

**Data set.** The dataset came from the combined cycle power plant dataset (the open power dataset), which contains 9568 data samples collected over six years from 2006 to 2011. The features include the average temperature of the environment per hour, the average pressure of the environment per hour, the average relative humidity of the environment per hour, the exhaust vacuum per hour, and the predicted label, which is the net energy output per hour. To simulate online data streams, we input these samples in batches in accordance with the strategy shown in Figure 1. We performed normalization on all sample values, resulting in the feasible range of features and labels being [0, 1]. This normalization process ensured consistency in the range of values for both features and labels.

**Basic parameters settings.** In the experiments, we poisoned stream $D_{s}$ at 5%, 10%, 15% and 20% poisoning rates. In previous work, poisoning rates higher than 20% were only rarely considered, as the attacker was typically assumed to be able to control only a small fraction of the training data [19]. The termination condition, ε, for algorithm convergence was set to 0.001. The decay parameter, α, for updating feature values of the poisoned sample points in the direction of gradient ascent was set to 0.01. The above parameter settings referred to those in [21].

The following chapters are organized according to those five questions above, with each section corresponding to one question.

### 5.1. Effectiveness Comparison of Online and Offline Poisoning Attacks

In this experiment, we divided 9568 sample points into 10 parts and sequentially input them into the ODPA-SP algorithm to simulate a scenario in which points of the data stream arrived at the cache one by one. Power data samples exhibited a stronger time series relationship. Therefore, in this experiment, the order of samples was maintained and the same order was used for each simulation training procedure. This approach ensured that the temporal relationship between the power data samples was preserved during the training process, which was critical for achieving accurate and reliable results. After 10 attacks, we recorded the time and loss of each attack, and calculated the average loss and the total time of all executions. We also input all 9568 sample points into the ODPA-SP algorithm at once to simulate the offline poisoning attack scenario, recording the attack time and loss. As shown in Table 3, the total time of the ten attacks was 17.37 s, which is less than half of the time of the offline attack, while the average loss caused by the ten attacks was comparable to that of the offline attack. The results indicate that the online attack method can significantly reduce time overhead while maintaining poisoning effectiveness.

### 5.2. Performance of Point Selection Strategy

**Figure 5:**
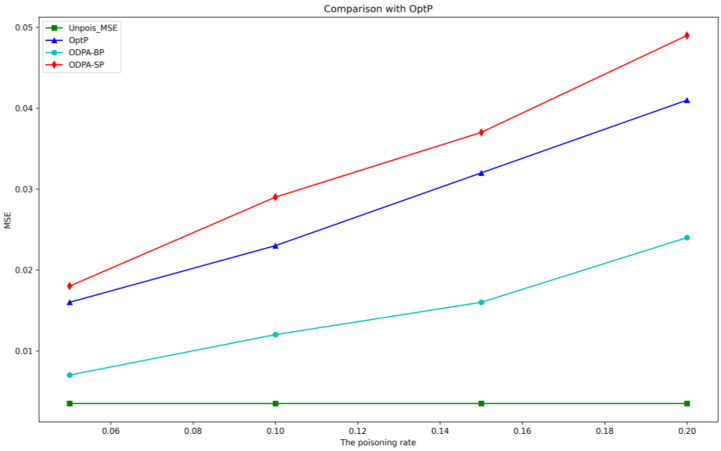
MSE comparison of attacks.

In this section, we selected 1125 sample points and tested the ODPA-SP, ODPA-BP and OptP at different poisoning rates. The experimental results are shown in Table 4 and Figure 5.

It is observed that all three attacks can mislead the predictive performance of linear regression models, and the change in MSE is also linear and upward with the increase in poisoning rates. The red line in Figure 5, representing the ODPA-SP algorithm, shows the best performance with the highest loss, which demonstrates the effectiveness of the point selection strategy. Specifically, the single-point poisoning strategy (ODPA-SP) outperforms the batch-points strategy (ODPA-BP) in terms of model loss function values after attack. This is because the former takes more time to select discontinuous data points one by one for poisoning in exchange for better results, while the latter selects continuous sub-sequent poisoning samples to optimize the objective function and reduce the computation time. The next section of experiments further confirms the time difference between ODPA-SP and ODPA-BP.

### 5.3. Performance of Batch-Poisoning Strategy

In this section, we conducted experiments on sample sets with 285, 1125, and 9568 data points at four poisoning rates for each of the three algorithms, and record the execution time. The results as Table 5 show that although the ODPA-BP algorithm does not cause a high MSE, it significantly reduces the execution time compared to the other two algorithms. This demonstrates the effectiveness of the batch-poisoning strategy in reducing time overhead of attack.

### 5.4. Comparison between ODPA-SP and ODPA-BP

**Figure 6:**
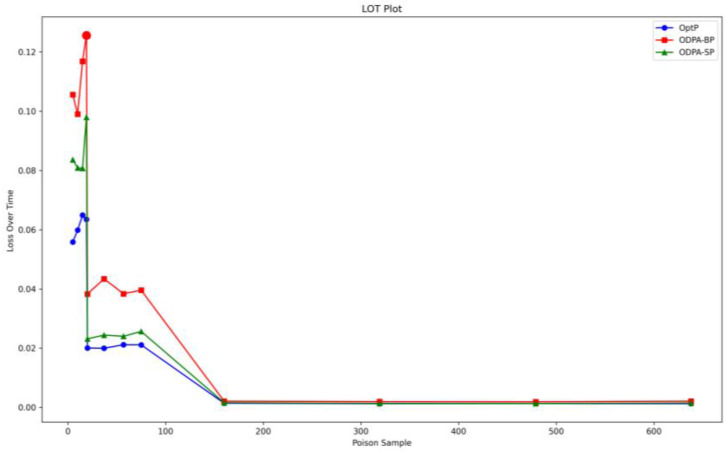
LOT competition of attacks.

**Figure 7:**
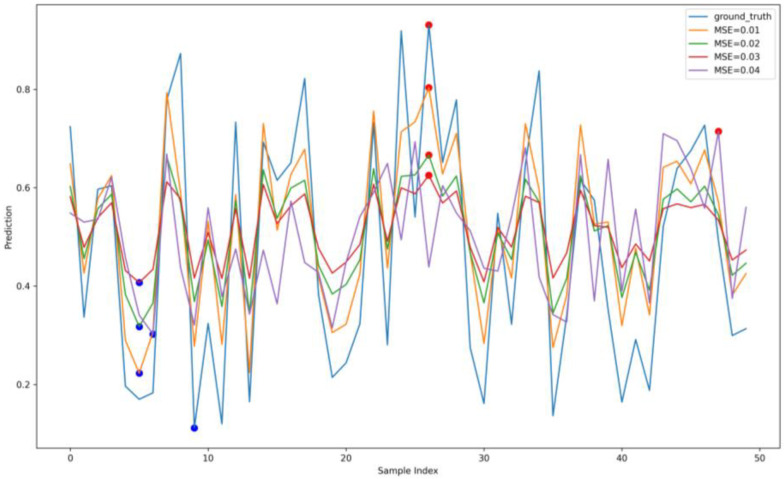
Impact of poisoning attacks on power prediction.

Regarding question 4, we conducted a detailed analysis of the MSE, time, and LOT of the three algorithms, based on the experimental settings described in the previous section. The results are presented in Table 6, and the comparative values of LOT are plotted in Figure 6 (where the horizontal axis represents the total number of poisoned samples and the vertical axis represents the LOT index). The analysis indicates that the selection of attack algorithms should not be limited to the degree of improvement in MSE alone, but should consider LOT comprehensively. Figure 6 clearly shows the performance comparison of the three algorithms in the LOT index, with the peak value of the red line being significantly higher than that of the other two lines. This indicates that although the ODPA-BP algorithm performs only moderately effectively in the MSE index, its comprehensive efficiency is optimal; in other words, it achieves the maximum MSE loss with the minimum time cost. In addition, Figure 6 also shows the trend of the performance of the three algorithms as the number of poisoned samples increased. All three algorithms achieved their maximum performance when the number of poisoned samples was around 57 (out of a total of 285 samples with a poisoning rate of 0.2), providing important guidelines for setting the optimal cache size. Table 7 shows the changes in average loss of the three algorithms with respect to the number of catch samples when the poisoning rate was fixed at 0.2. We can observe that when the number of samples was less than 285, the model did not reach a stable state due to the insufficient number of samples, which is reflected in the large fluctuations in the loss values of the model for the clean samples. At this time, the loss values after the model was attacked were also unstable, and the attack had no practical significance. However, when the number of samples exceeded 285, the model reached a stable state, and the loss value of the model for the clean samples stabilized at around 0.0037. At this time, the loss value after the model was attacked stabilized at around 0.04, indicating that when the cache sample size was set to around 285, the attack algorithm reached the optimal state. This conclusion is consistent with the conclusions of Table 6 and Figure 7.

### 5.5. Actual Impact of Poisoning Attacks on Power Prediction

The effectiveness of attacks introduced in the previous section is reflected only in data indicators such as MSE, which does not directly show their true impact on the power system. This section will directly present the relationship between the MSE index and the predicted results of electric energy, as shown in Figure 7. The horizontal axis in Figure 7 represents the number of prediction samples, and the vertical axis represents the predicted electric energy (the values shown are not the actual values of electric energy, but the normalized results that can still reflect the real data status). The blue line represents the ground truth of electric energy, while the other colored lines represent the predicted values under different MSE. The figure shows that a significant deviation occurs between the predicted energy values generated by attacks and the ground truth. The red and blue solid circles represent the maximum deviation of the predicted values, which becomes more severe as the increase in the MSE. Typically, the deviation of electric energy prediction must be controlled within 5% to ensure sufficient safety. However, the deviation shown in the figure has already exceeded this threshold. If not intervened, it will lead to overestimation or underestimation of the predicted electric energy, causing serious imbalance in the power grid load. Therefore, the purpose of this experiment is to demonstrate the necessity of defending against poisoning attacks.

### 5.6. Other Questions

In this paper, the setting of parameters such as the sliding window size, m, for batch-points selection, and the number of samples being covered, β, are not extensively discussed. We set m to the number of poisoned samples, considering the extreme case of overall contamination of the poisoning points. This setting could reduce the number of iterations and minimize the time cost. Secondly, for the setting of the forgetting parameter of β, we set β=L, also considering the extreme case of complete forgetting. The experimental results are still quite promising. This paper focuses on feasibility and effectiveness, and optimal parameter settings will be completed in future research.

## 6. Conclusions

This paper addresses the problem of poisoning online regression in the edge computing environment of the smart grid for the first time. Specifically, we propose an online poisoning attack framework that transforms the bi-level optimization problem from the offline mode to the online mode. This is equivalent to converting a one-time processing of a massive offline sample into multiple batch processing. By optimizing the sample size processed in each batch, we could reduce the time overhead of each processing and achieve the goal of reducing the overall time overhead. Then, this paper applies the loss-based selection strategy and the batch-polluting strategy in poisoning attacks on regression models. Finally, we evaluate the proposed algorithms for the edge device with the data stream being generated using a simulation based on offline open datasets of the smart grid. Our experiments have shown that the proposed method can reduce time overhead by over 50% while also improving average attack effectiveness by more than 1.23 times. The results emphasize the importance of defending against poisoning attacks in the context of smart grid security.

To ensure timeliness, we focused on common online prediction models that are suitable for limited computing and resource-constrained environments in the edge intelligence environment. In future work, we plan to investigate more complex online models of deep learning and neural networks, which will enable us to explore poisoning attack and defense strategies in greater depth.

## Figures and Tables

**Table 1:** A summary of the major poisoning attack methodology.

Poisoning Strategy	Method Already Proposed	Poisoning Area	Adversary’s Knowledge	Online/Offline	Classification/Regression
Label flipping	InvFlip [19], ALFA [29], LFA [30], and BFlip [19]	***y***	Black-box	Offline	Classification
Gradient ascent	[31,33,34,35,38]	(***X***,***y***),***X***	Black-box and White-box	Offline	Classification
	OptP [19] and BGD [19,43]				Regression
	[42]	***X***	White-box	Online	Classification
Statistical-based	StatP [19]	(***X***,***y***)	Black-box	Offline	Regression
General optimal control	[41]	***X***	Black-box	Online	Classification

**Table 2:** Notation description.

Symbol	Description
y	The label vector of the sample
***X***	The feature matrix of the sample, where k represents the feature dimension and n represents the total number of samples
θ	The parameter vector of the regression model
h(X)	The objective function of the learning algorithm
$J\left(D_{s},\theta \right)(abbreviated\;as\;J)$	The loss function of the mean square error
$D_{s}={\left\{(x_{i},y_{i})\right\}}_{i=1}^{n}$	The dataset containing n samples, where each sample consists of a feature vector, $x_{i}$, and a corresponding label, $y_{i}$
$argminJ(D_{s},\theta )$	Using $D_{s}$ to train the model to minimize the loss function
$\theta^{*}$	The global optimal parameter vector
$\nabla_{\theta_{i}}J(D_{s},\theta_{i})$	The gradient of loss function with respect to the parameter
α	The learning rate in the gradient algorithm
*β*	The number of samples being covered
*L*	The capacity size of the cache
t	The time t
$D_{s}^{\left(t\right)}$	The partial training samples of time t
$n_{p}^{\left(i\right)}$	The poisoned sample number of time t
γ	The poisoning rate
L	The learning algorithm
$\theta^{\left(t\right)}$	The parameter of time t
ε	The termination condition epsilon (default = 0.001)
Π	The projection operator
$\nabla_{x_{c}^{\left(t\right)}}J(D_{s}^{\left(t\right)},\theta^{\left(t\right)})$	The gradient of the loss function with respect to $x_{c}$ at time t,
$\nabla_{x_{1:q}^{\left(t\right)}}J(D_{s}^{\left(t\right)},\theta^{\left(t\right)})$	The gradient of the loss function with respect to the batch points $x_{1}$ to $x_{q}$ at time t, where q represents the number of poisoning sample points.
m	Width of slide window during batch point selection

**Table 3:** Comparison of online and offline poisoning attacks.

Cache	Cache_1	Cache_2	Cache_3	Cache_4	Cache_5	Cache_6	Cache_7	Cache_8	Cache_9	Cache_10
**MSE**	0.04402040	0.04428521	0.04931959	0.03914929	0.04085501	0.04801267	0.04876293	0.04010114	0.04511061	0.04883171
**Time**	1.699955	1.642623	1.677615	1.869981	2.021997	1.68938	1.710946	1.692828	1.674517	1.692794
	**Average_Test_MSE (Online)**									**0.044844856**
	**Total time of 10 caches (Online)**									**17.372636 s**
	**MSE of 9568 (Offline)**									0.04484707
	**Total time of 9568 (Offline)**									34.713239 s

**Table 4:** MSE comparison of attack effect between loss-based point selection and random point selection.

The Poisoning Rate	MSE				Sample Location	
	Unpoisoned_MSE	OptP	ODPA-BP	ODPA-SP	Single	Batch
0.05	0.0035	0.016	0.007	0.018	83,49,30,2,51	41,42,43,44,45
0.1	0.0035	0.023	0.012	0.029	83,49,30,2,51, 43,27,91,25,85	41,42,43,44,45, 46,47,48,49,50
0.15	0.0035	0.032	0.016	0.037	83,49,30,2,51, 43,27,91,25,85, 73,11,79,93,31	6,7,8,9,10, 11,12,13,14,15, 16,17,18,19,20
0.2	0.0035	0.041	0.024	0.049	83,49,30,2,51, 43,27,91,25,85, 73,11,79,93,31, 5,63,55,39,59	6,7,8,9,10, 11,12,13,14,15, 16,17,18,19,20, 21,22,23,24,25, 26,27,28,29,30

**Table 5:** Time comparison of three attacks.

Sam_Num	Pois_Rate	Time(s)		
		OptP	ODPA-BP	ODPA-SP
285	0.05	0.286477	**0.056842**	0.19136
	0.1	0.41782	**0.080812**	0.284599
	0.15	0.477785	**0.102725**	0.346848
	0.2	0.59849	**0.119408**	0.388027
1125	0.05	0.798881	**0.182836**	0.779118
	0.1	1.153583	**0.276719**	1.188495
	0.15	1.513819	**0.417298**	1.542514
	0.2	1.942288	**0.606962**	1.911988
9568	0.05	12.381818	**4.346872**	12.280106
	0.1	19.376246	**6.738431**	18.720726
	0.15	26.736512	**9.535606**	26.131643
	0.2	34.670963	**12.059491**	34.033881

**Table 6:** Comparative study of three attacks.

Pois_Num	Time(s)			MSE			Loss_Over_Time (LOT)		
	OptP	ODPA-BP	ODPA-SP	OptP	ODPA-BP	ODPA-SP	OptP	ODPA-BP	ODPA-SP
15	0.286477	0.056842	0.19136	0.016	0.006	0.016	0.055850906	0.105555751	0.08361204
29	0.41782	0.080812	0.284599	0.025	0.008	0.023	0.059834378	0.098995199	0.080815463
43	0.477785	0.102725	0.346848	0.031	0.012	0.028	0.06488274	0.116816744	0.080727004
57	0.59849	0.119408	0.388027	0.038	0.015	0.038	**0.063493124**	**0.125619724**	**0.09793133**
81	0.798881	0.182836	0.779118	0.016	0.007	0.018	0.020028014	0.038285677	0.023103047
113	1.153583	0.276719	1.188495	0.023	0.012	0.029	0.01993788	0.043365291	0.024400607
169	1.513819	0.417298	1.542514	0.032	0.016	0.037	0.021138591	0.038341904	0.023986816
225	1.942288	0.606962	1.911988	0.041	0.024	0.049	0.021109125	0.03954119	0.025627776
478	12.381818	4.346872	12.280106	0.017	0.009	0.019	0.001372981	0.002070454	0.001547218
957	19.376246	6.738431	18.720726	0.024	0.013	0.025	0.00123863	0.001929232	0.001335418
1435	26.736512	9.535606	26.131643	0.034	0.018	0.034	0.001271669	0.001887662	0.001301105
1914	34.670963	12.059491	34.033881	0.043	0.025	0.050	0.001240231	0.002073056	0.001469124

**Table 7:** Change in loss with sample at fixed poisoning rate.

Sam_Num	Unpois_MSE	Test_MSE
16	0.0176	0.554
76	0.0039	0.053
100	0.0046	0.045
189	0.0028	0.045
**285**	**0.0035**	**0.039**
376	0.0035	0.041
1125	0.0037	0.037
1876	0.0038	0.041
3750	0.0036	0.041
9568	0.0036	0.042

## Data Availability

Publicly available datasets were analyzed in this study. This data can be found here: [https://archive.ics.uci.edu/ml/machine-learning-databases/00294/CCPP.zip], accessed on 26 February 2023. The dataset after normalization preprocessing can be downloaded from the code link provided in Section 5 of this paper. Data citation: [dataset] Tüfekci, P.; Kaya, H. 2014. Combined Cycle Power Plant Data Set; UCI Machine Learning Repository [https://archive.ics.uci.edu/ml/]; Version 2014-03-26.
